# Sketching the Landscape of Speech Perception Research (2000–2020): A Bibliometric Study

**DOI:** 10.3389/fpsyg.2022.822241

**Published:** 2022-06-02

**Authors:** Juqiang Chen, Hui Chang

**Affiliations:** School of Foreign Languages, Shanghai Jiao Tong University, Shanghai, China

**Keywords:** bibliometric analysis, research productivity, speech perception, research collaboration, research trends

## Abstract

Based on 6,407 speech perception research articles published between 2000 and 2020, a bibliometric analysis was conducted to identify leading countries, research institutes, researchers, research collaboration networks, high impact research articles, central research themes and trends in speech perception research. Analysis of highly cited articles and researchers indicated three foundational theoretical approaches to speech perception, that is the motor theory, the direct realism and the computational approach as well as four non-native speech perception models, that is the Speech Learning Model, the Perceptual Assimilation Model, the Native Language Magnet model, and the Second Language Linguistic Perception model. Citation networks, term frequency analysis and co-word networks revealed several central research topics: audio-visual speech perception, spoken word recognition, bilingual and infant/child speech perception and learning. Two directions for future research were also identified: (1) speech perception by clinical populations, such as hearing loss children with cochlear implants and speech perception across lifespan, including infants and aged population; (2) application of neurocognitive techniques in investigating activation of different brain regions during speech perception. Our bibliometric analysis can facilitate research advancements and future collaborations among linguists, psychologists and brain scientists by offering a bird view of this interdisciplinary field.

## Introduction

Speech perception is a vital means of human communication, which involves mapping speech inputs onto various levels of representation, for example phonetic/phonemic categories and words (Samuel, 2011). However, the underlying mechanism of this process is more complex than it appears to be. First, unlike a written sequence of words, the speech stream is continuous and thus requires segmentation before it can be further processed. Second, there is no simple or direct correspondence between phones/phonemes as perceived and the acoustic patterns generated by articulatory gestures due to variations induced by different phonetic contexts and talker vocal characteristics.^1^ The fact that adult native speakers can effortlessly perceive speech from various speakers even in relatively noisy environments has intrigued researchers from multiple disciplines, such as audiology, experimental psychology, brain science, computer science, phonetics and linguistics.

Historically speaking, compared with other aspects of human perception, the study of speech perception began relatively late around the 1950s thanks to the accessibility of technologies that are essential for speech research, such as sound spectrographs and acoustic speech synthesisers. In one historical review, Hawkins divided speech perception research into three periods (Hawkins, 2004): the early period (1950–1965), the middle period (1965–1995) and recent developments (1995–2004). The early period saw attempts to examine some fundamental issues in speech perception, such as cerebral dominance in speech processing (Kimura, 1961), the role of memory and context, the separation of a speech signal from environment sounds or integration of different modalities (Sumby and Pollack, 1954). The second period was built on the first period and witnessed birth and growth of important research topics, for example categorical perception, that is listeners perceive categories when presented with continuous stimuli, and theories, for example the motor theory (Liberman et al., 1967) and the quantal theory (Stevens, 1972). Although the review provided insightful reflections on key issues and achievements of the first two stages, it did not cover much of the advancements in the twenty-first century. There exist several other recent qualitive reviews of speech perception (Diehl et al., 2004; Samuel, 2011; Antoniou, 2018); however, they focused more on general theoretical aspects of the field with consideration of only a limited number of research articles.

The first two decades of the twenty-first century have witnessed a surge of academic publications in all fields. It is, thus, increasingly challenging to keep oneself at the front line of and to have a bird view of research developments, especially in interdisciplinary fields, such as speech perception. Synthesising research findings effectively from different disciplines becomes an integral part of research advancements. Bibliometric analysis provides a systematic, transparent and objective approach to understanding a certain research field (Aria and Cuccurullo, 2017). It employs statistical and data visualisation methods to examine bibliographic big data, covering a much larger scope of research items compared with traditional qualitative reviews. So far, the field of speech perception has not been investigated *via* bibliometric analysis, although there exist such attempts in neighbouring fields, such as linguistics (Lei and Liao, 2017), applied linguistics (Lei and Liu, 2019), and in some interdisciplinary topics, such as visual word recognition (Fu et al., 2020), second language pronunciation (Demir and Kartal, 2022) and multilingualism (Lin and Lei, 2020).

In the present study, a bibliometric analysis was conducted to answer the following research questions:

1. What are the most active countries and research institutes, who are the leading researchers, and how do they collaborate in speech perception research?
2. What are the most impactful theories and models?
3. What are the important research themes/topics and future directions?

## Materials and Methods

### Data Collection

First, we did a topic search of the term ‘speech perception’ (in the quotation mark) using the Web of Science Core Collection^2^ for research articles (Document type = article) published in journals indexed in Social Sciences Citation Index (SSCI), Science Citation Index Expanded (SCIE), Emerging Sources Citation Index (ESCI) on 24th of February 2021. The time span was set between 2000 and 2020 and non-English papers were excluded. Originally, 9,436 research articles were found. Given that the focus of the present bibliometric analysis is on speech perception from the perspectives of phonetics/linguistics, psychology, neuroscience and speech pathology, pure medical research under the Web of Science category of otorhinolaryngology (*n* = 2,731) was excluded.

**Figure 1:**
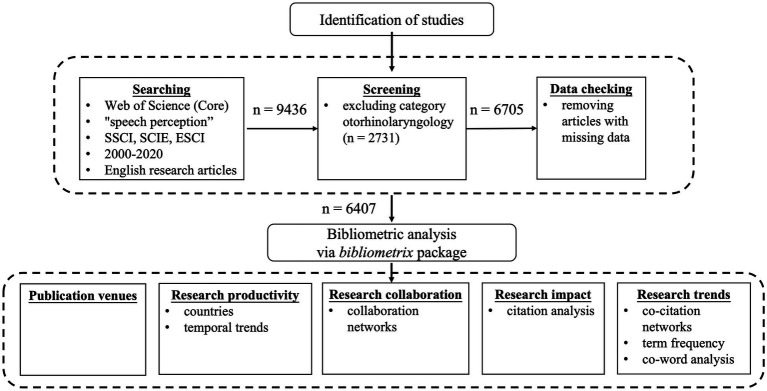
The workflow diagram.

The full records and cited references of these articles were downloaded and processed by the *bibliometrix* package (Aria and Cuccurullo, 2017) in *R* (R Core Team, 2018). We checked the data set and removed articles with missing data, which further reduced the number of research articles to 6,407 (see Figure 1). The *bibliometrix* package developed in the *R* language provides a variety of functions for comprehensive bibliometric analysis and can be integrated with other *R* packages seamlessly for more advanced data modelling and visualisation. We chose the *bibliometrix* package over other software because it provides a more open, flexible, customisable and reproducible workflow.

### Data Wrangling

The downloaded bibliometric information was converted to a data frame, in which each row represents one article and each column one field tag in the original export file. *Bibliometrix* automatically implemented a set of cleaning rules. First, all texts were transformed into uppercase. Second, non-alphanumeric characters, punctuation symbols and extra spaces were removed. Third, authors’ first and middle names were truncated to the initials.

## Results and Discussion

The present study surveyed a period of 21 years, and 6,407 journal articles that met our selection criteria were authored by 12,381 researchers, 438 of whom wrote single-authored articles and 11,943 of whom co-authored with others (see Table 1). 564 articles are single-authored and the number of authors for each paper on average is 1.93. The collaboration index, that is co-authors per article calculated only using the multi-authored article set, is 2.04, indicating that most speech perception research involves collaboration between at least two authors. As for research impact, the average number of citations per document is 27.91 times and that breaks down to 2.42 times per year.

**Table 1:** Summary information on retrieved speech perception studies (2000–2020).

Descriptions	Counts and rates
Documents	6,407
Average citations per document	27.91
Average citations per year per doc	2.42
References	129,940
Keywords plus	7,501
Author’s keywords	8,333
**Authors**	
Authors	12,381
Author appearances	23,242
Authors of single-authored documents	438
Authors of multi-authored documents	11,943
**Authors Collaboration**	
Single-authored documents	564
Documents per author	0.52
Authors per document	1.93
Co-Authors per documents	3.63
Collaboration index	2.04

In the following sections, we first report major publication venues for speech perception research (section “The Most Popular Publication Venues”) and then investigate research productivity as indicated by publications and identify leading countries and researchers (section “Research Productivity”). Next, we demonstrate connections among universities/research institutes and countries *via* collaboration networks (section “Research Collaborations”). Last but not least, foundational and time-honoured theories of speech perception were revealed *via* citation analysis of publications and authors, whereas co-citation networks, bibliographic coupling networks, term frequency and co-word analysis based on keywords and abstracts were used to uncover more recent research themes/cohorts and future directions (section “Impactful Research Work and Key Research Themes”).

### The Most Popular Publication Venues

Table 2 shows the top 20 journals that publish speech perception studies. These journals belong to different disciplines, indicating the interdisciplinary nature of the field. For example, *Journal of the Acoustical Society of America* published the largest number of speech perception research, and two subjects in its scope that are relevant to speech perception are speech, music and noise, as well as psychology and physiology of hearing. *Developmental Science* published 76 papers on this topic from the perspective of developmental psychology. Other publication channels range from linguistics/phonetics journals (e.g., *Journal of Phonetics, Language and Speech*), to psychology or psycholinguistic journals (e.g., *Journal of Speech Language and Hearing Research*, *Frontiers in Psychology, PLOS one, Journal of Experimental Psychology*), as well as neuroscience journals (e.g., *Neuroimage, Brain and Language, Neuropsychologia, Journal of Neuroscience, Frontiers in Human Neuroscience, Cerebral Cortex, Journal of Cognitive Neuroscience, Neuroreport*).

**Table 2:** Top 20 journals publishing speech perception studies.

Sources	Articles	Articles per year
Journal of the Acoustical Society of America	443	22.15
Journal of Speech Language and Hearing Research	360	18.00
Frontiers in Psychology	232	17.8
Plos One	190	13.57
Neuroimage	155	7.75
Brain and Language	150	7.50
Journal of Phonetics	145	7.25
Neuropsychologia	132	6.60
Cognition	131	6.55
Journal of Experimental Psychology-Human Perception and Performance	100	5.00
Journal of Neuroscience	99	4.95
Speech Communication	98	4.90
Attention Perception & Psychophysics	94	4.70
Frontiers in Human Neuroscience	82	6.30
Journal of Memory and Language	81	4.05
Developmental Science	74	3.70
Language and Speech	74	3.70
Proceedings of the National Academy of Sciences of The United States of America	74	3.70
Cerebral Cortex	71	3.55
Neuroreport	70	3.50

It should be noted that the journal rankings based on the total number of publications should be interpreted with caution as some new journals, that is *PLOS one* (2006), *Frontiers in psychology* (2007) and *Frontiers in human neuroscience* (2007), were launched after 2000. Thus, we also normalised the total publications by the years of publication during the period of survey, for example 14 years for *PLOS one*. The rankings for *PLOS one* and *Frontiers in Psychology* did not change but that for *Frontiers in Human Neuroscience* changed.

### Research Productivity

**Figure 2:**
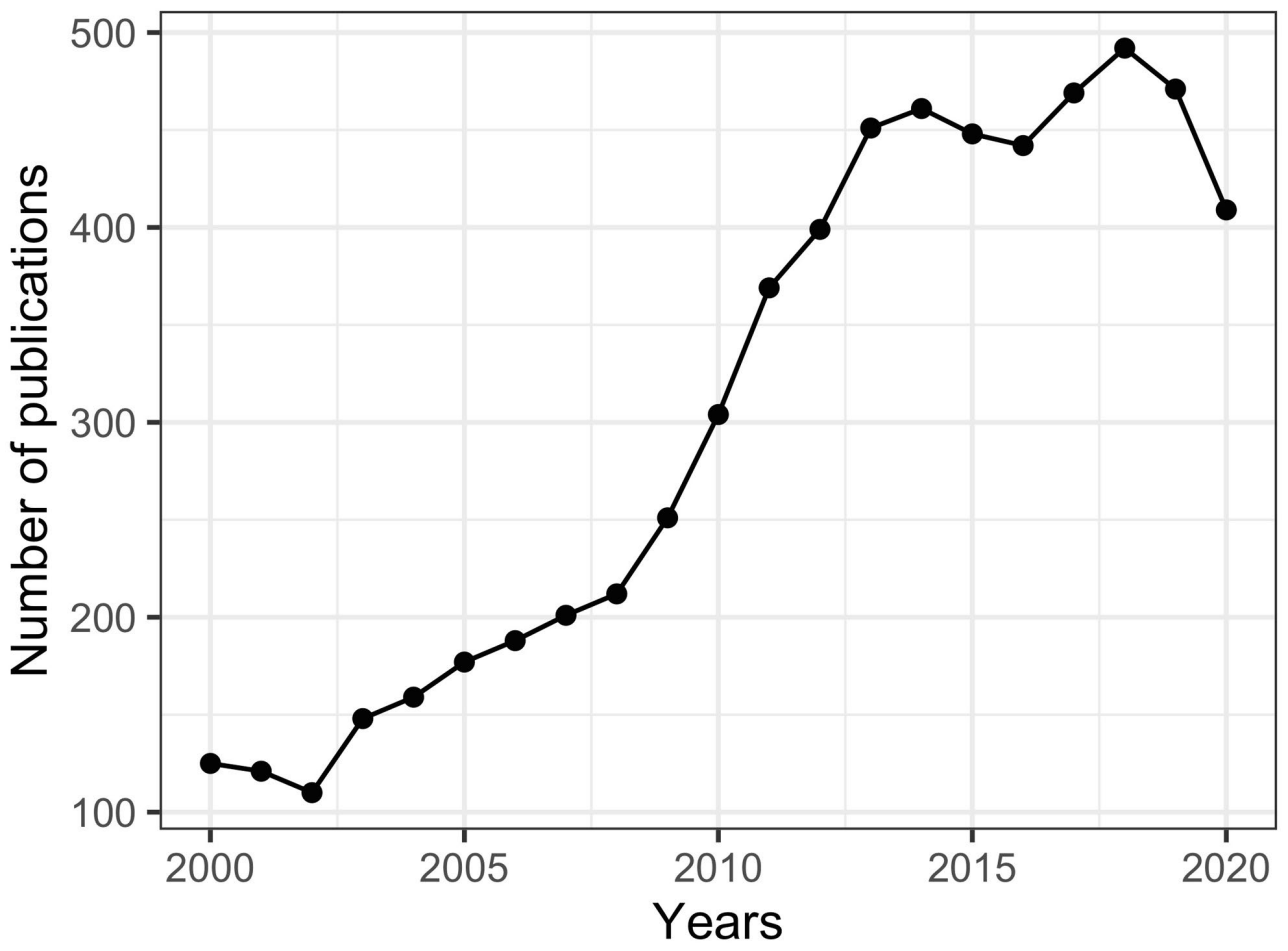
Publications per year of speech perception studies (2000–2020).

There is a yearly and drastic increase in speech perception journal articles from 2000 to 2020 (see Figure 2), suggesting that it is an area of increasing productivity. A drop can be seen in 2020, reflecting the impact of the COVID-19 pandemic. Given that most speech perception experiments require face-to-face testing, social distancing requirements slowed down or interrupted some of the data collection activities and led to a short-term deceleration in research output.

To address this issue, it should be noted that many online testing environments and software had been developed before COVID-19 for psychological experiments and they yield generally comparable results to the data obtained in the lab (Crump et al., 2013) if users follow standardised and widely accepted guidelines (Grootswagers, 2020). It is expected that these online testing methods will be further developed, better recognised and more frequently applied than before the pandemic.

In addition, publication counts were calculated for each country and the top ten most productive countries were listed in Table 3. The United States is undoubtedly the leading country in this area of research, followed by the United Kingdom. It is also interesting to note that multiple country publications account for a relatively small portion of all articles published by US researchers, suggesting that a relatively large amount of research work was done through domestic collaboration. On the other hand, France, China and Spain have a higher proportion of multiple country publications, although their total number of international publications was not as high as the United States and the United Kingdom.

**Table 3:** Top 10 most productive countries in speech perception research (2000–2020).

Country	Articles	SCP	MCP	MCP/Total ratio (%)
USA	2,444	2074	370	0.15
United Kingdom	574	405	169	0.29
Canada	462	306	156	0.34
Germany	399	255	144	0.36
France	310	163	147	0.47
Netherlands	301	187	114	0.38
China	262	151	111	0.42
Australia	251	160	91	0.36
Japan	161	129	32	0.20
Spain	136	73	63	0.46

SCP, Single Country Publications; MCP, Multiple Country Publications.

**Figure 3:**
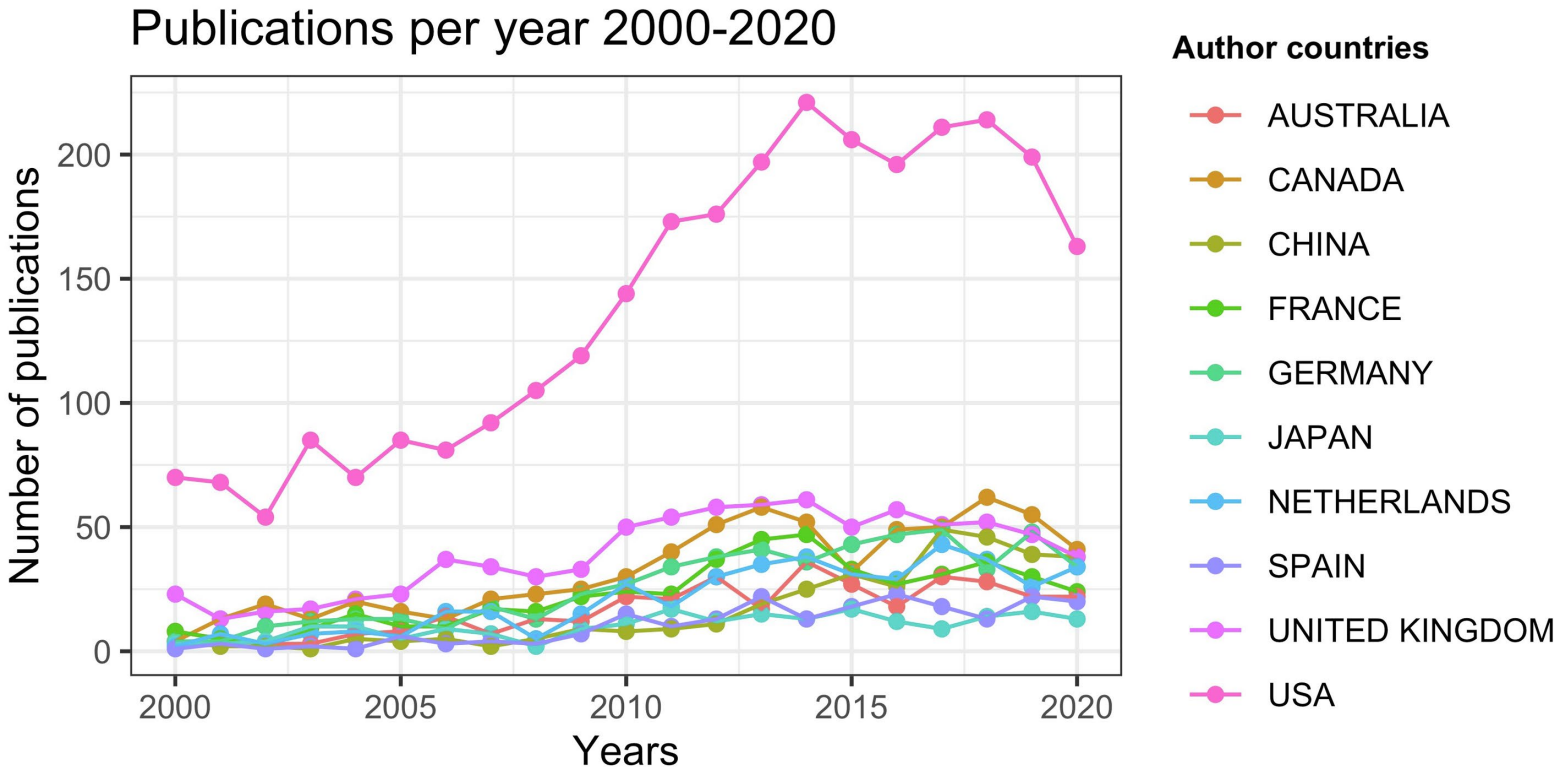
Publication trends in speech perception research for the top 10 most productive countries.

To examine variations in research productivity for each of the ten countries over the 21-year period, we plotted the growth curve for each of them in Figure 3. The United States is the leading country in the number of publications throughout the period with an overall higher increase rate than the other nine countries, which have relatively low increase rates.

The top 20 most productive authors are shown in Table 4. Mitterer H. came first in terms of number of journal articles, followed by McQueen J.M. Both authors also have a high H-index, a measure of research impact, that is the highest number of publications of a scientist that each received *h* or more citations (Schreiber, 2008). Pisoni D.B., Rosen S., Kraus N. and Werker J.F. also have published more than 40 papers with relatively high H-index.

**Table 4:** Top 20 most productive authors of speech perception research (2000–2020).

IDs	Authors	No. of publications	H-index	Total Citations
1	Mitterer H.	44	22	1,124
2	McQueen J. M.	42	18	956
3	Pisoni D. B.	41	23	1,944
4	Rosen S.	41	22	3,177
5	Kraus N.	40	29	3,232
6	Werker J. F.	40	23	2,865
7	McMurray B.	39	21	1,671
8	Schwartz J. L.	37	15	787
9	Holt L. L.	36	20	931
10	Hugdahl K.	33	21	1,280
11	Sato M.	32	15	660
12	Ackermann H.	31	18	1,219
13	Escudero P.	31	14	660
14	Hickok G.	31	22	2,002
15	Soto-Faraco S.	31	15	842
16	Nittrouer S.	30	14	680
17	Samuel A. G.	30	15	1,159
18	Bidelman G. M.	28	13	658
19	Poeppel D.	28	19	3,503
20	Stevenson R. A.	28	18	1,292

**Figure 4:**
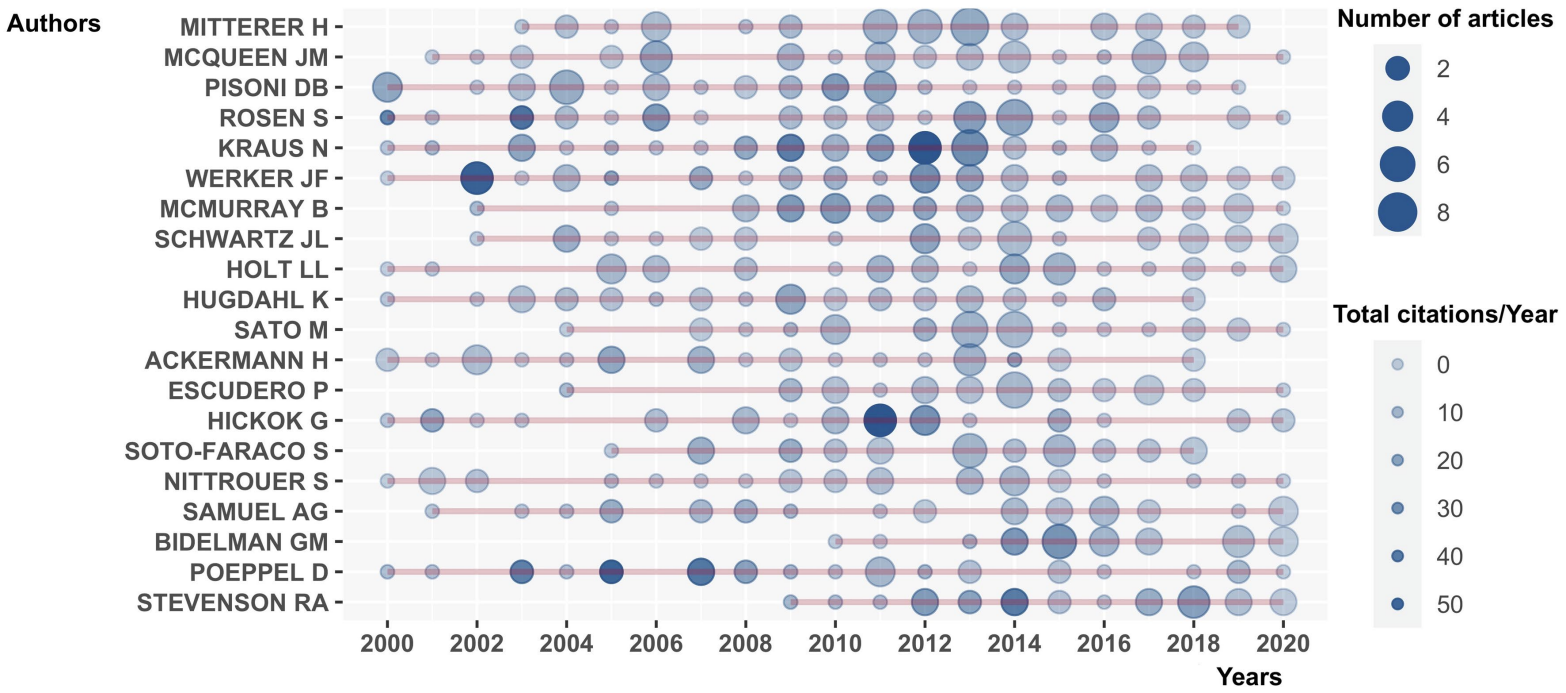
Yearly publications and citations for the top 20 productive researchers of speech perception (2000–2020). The size and the darkness of the circles are in proportion to the number of publications and to the total citations per year, respectively.

To characterise the dynamic relations between publication and citation, the yearly publication and citation counts were plotted in Figure 4. The size of the circles is in proportion to the number of publications. The darkness of the circles equals to the total citations per year. Authors, such as Rosen S., Kraus N., Werker J.F., Hickok G., Poeppel D. and Stevenson R.A., produced research work that were highly cited over the period from 2000 to 2020.

### Research Collaborations

Given that speech perception is an interdisciplinary topic that requires teamwork across traditional disciplines, and across universities/institutes and countries, collaboration networks were constructed here to demonstrate how authors, their affiliated institutions and countries related to each other.

**Figure 5:**
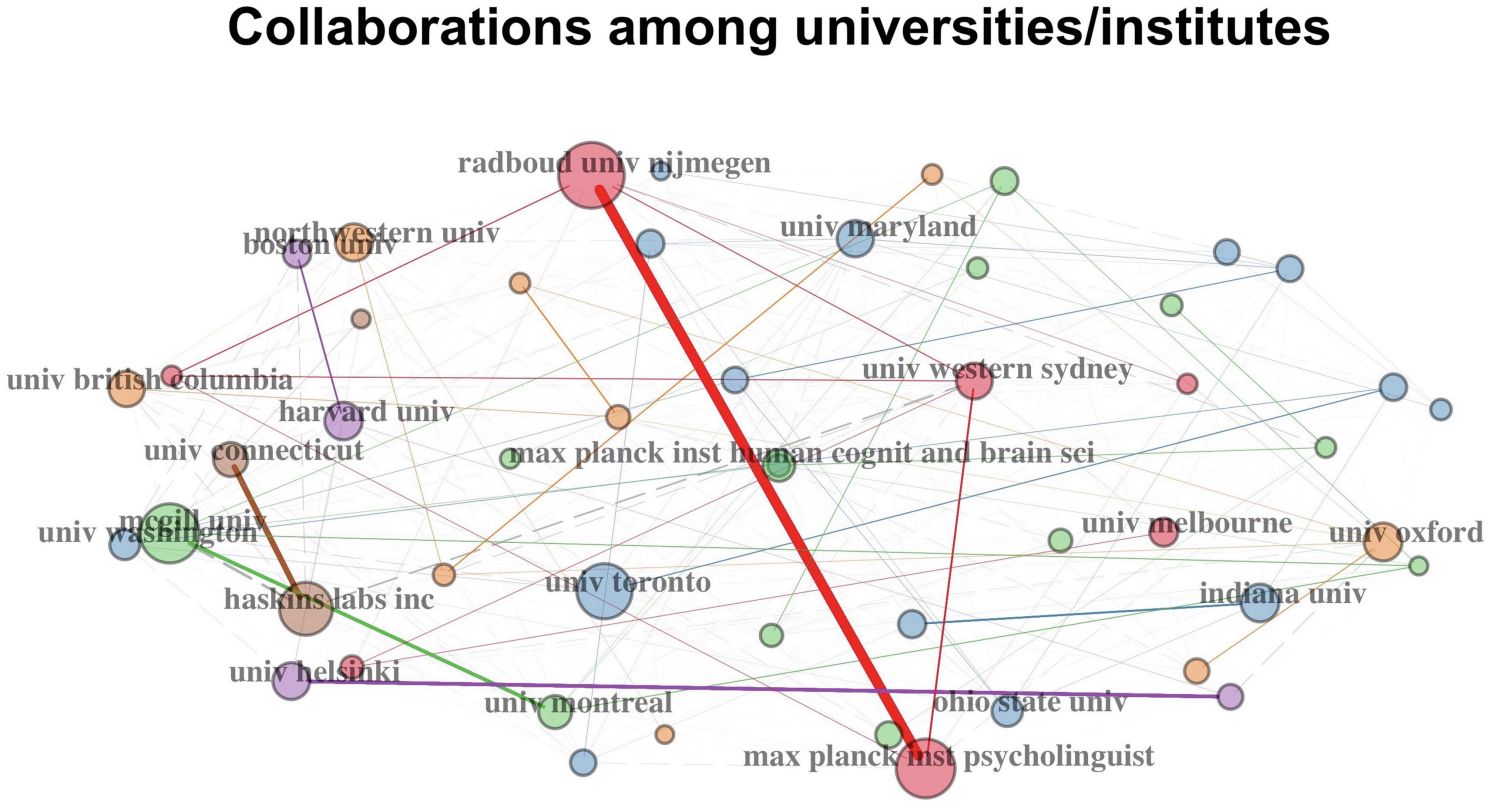
Top 20 active universities/institutes in speech perception research and their collaboration network. Each node in the network represents a different university/institute and the node’s diameter corresponds to the strength of the institute’s collaboration with others. Lines represent collaboration pathways among these universities or institutes.

Figure 5 illustrates the collaboration network of the top 20 active universities/institutes. The size of each node is proportional to its degree of collaboration. One prominent mega-cluster is the triangle collaboration pathway among Radboud University Nijmegen, the Max Planck Institute for Psycholinguistics and Western Sydney University. This was driven by the well-known psycholinguist, Anne Culter, former director of the Max Planck Institute for Psycholinguistics, who worked at both places over the period. Other clusters indicate research collaborations among universities in the United States, Canada and the United Kingdom.

**Figure 6:**
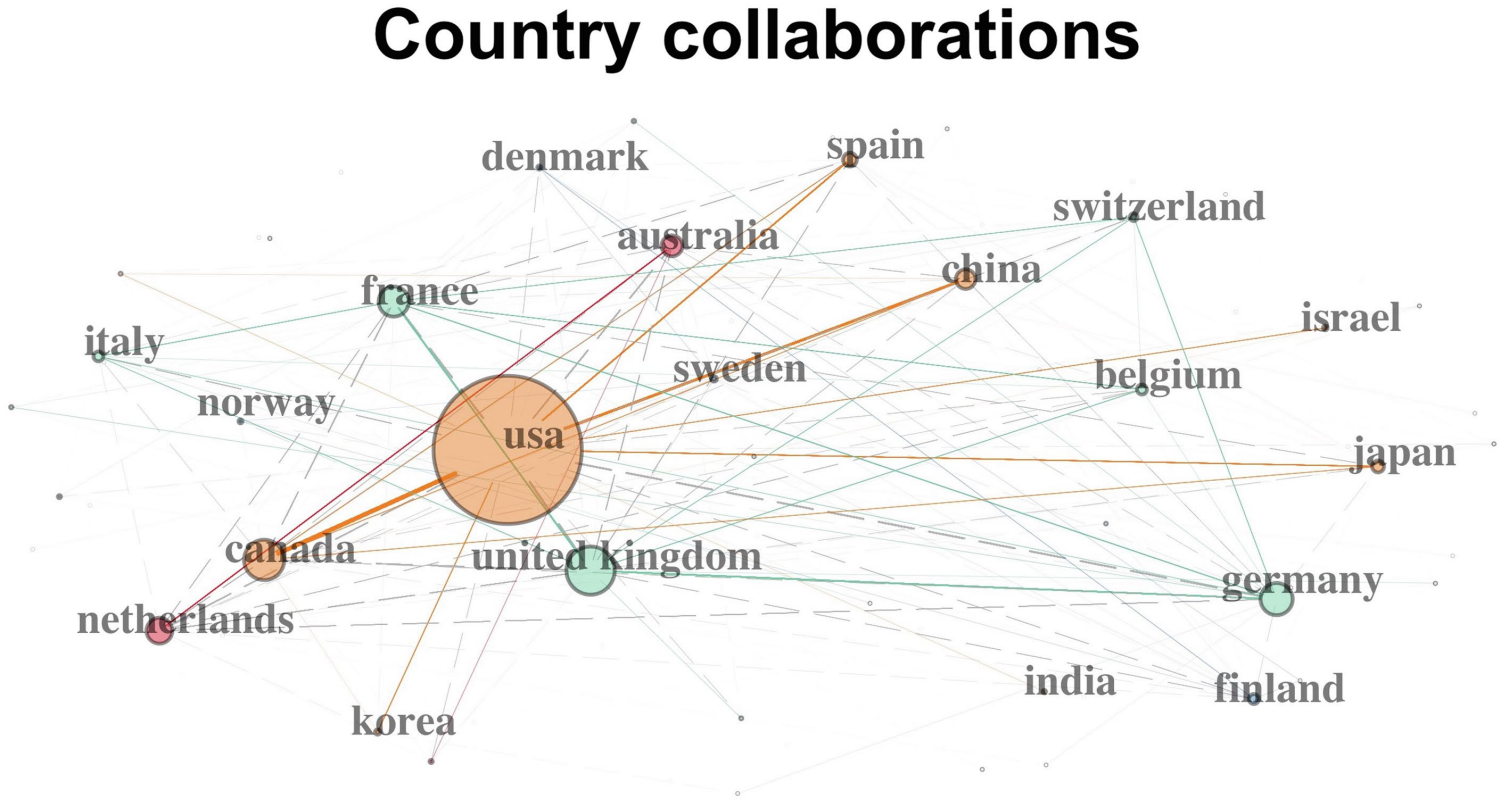
Top 20 countries’ collaboration on speech perception studies. Each node in the network represents a different nation and the node’s diameter corresponds to the strength of a nation’s collaboration with other countries. Lines represent collaboration pathways between countries.

Figure 6 shows the collaboration network of the top 20 collaborative countries. The United States has demonstrated a relatively strong collaboration capacity indicated by node size. Its collaboration pathways reached out to Canada, Spain, Japan and China. Another cluster connected some geographically close European countries, such as the United Kingdom, France, Germany, Belgium, Switzerland, Northway and Italy. A third pathway bridged the Netherlands and Australia, presumably for the same reason underlying the university-level collaboration as identified above between the two countries.

### Impactful Research Work and Key Research Themes

In the previous sections, we have identified key publication venues, leading researchers, universities and countries as well as their collaboration pathways. Next, we first uncover impactful research work and researchers *via* citation analysis and then identify major research themes/trends *via* citation network analysis and term frequency analysis of keywords, titles and abstracts as well as their co-occurrence patterns.

Table 5 shows high impact research as indicated by citations based on the articles in our data set as well as their cited references. The paper with the highest citation (McGurk and Macdonald, 1976), for example, reported the multisensory illusion in audio-visual speech perception, later also called the McGurk effect and the paradigm they used in the paper has a substantial influence on later research. Research item 3 (Sumby and Pollack, 1954) was a similar and even earlier attempt at examining visual contribution to speech perception, which found that the visual contribution to speech intelligibility negatively correlated with the speech-to-noise ratio. Both phenomena reflect the multi-modal nature of speech perception, which is an important theoretical issue.

**Table 5:** Top 20 highly cited research articles.

IDs	Researchers	Years	Sources	DOIs	Citations
1	McGurk H.	1976	Nature	10.1038/264746A0	582
2	Werker J. F.	1984	Infant Behav. Dev.	10.1016/S0163-6383(84)80022-3	498
3	Sumby W. H.	1954	J. Acoust. Soc. Am.	10.1121/1.1907309	449
4	Hickok G.	2007	Nat. Rev. Neurosci.	10.1038/NRN2113	441
5	Liberman A. M.	1985	Cognition	10.1016/0010-0277(85)90021-6	438
6	McClelland J. L.	1986	Cognitive Psychol.	10.1016/0010-0285(86)90015-0	397
7	Oldfield R. C.	1971	Neuropsychologia	10.1016/0028-3,932(71)90067-4	389
8	Liberman A. M.	1967	Psychol. Rev.	10.1037/H0020279	328
9	Shannon R. V.	1995	Science	10.1126/SCIENCE.270.5234.303	305
10	Kuhl P. K.	1992	Science	10.1126/SCIENCE.1736364	298
11	Goldinger S. D.	1998	Psychol. Rev.	10.1037/0033-295X.105.2.251	272
12	Tallal P	1980	Brain Lang.	10.1016/0093-934X(80)90139-X	231
13	Best C. T.	1995	Book Chapter^*^	See references	224
14	Eimas P. D.	1971	Science	10.1126/SCIENCE.171.3968.303	224
15	Flege J. E.	1995	Book Chapter	See references	219
16	Norris D	2003	Cognitive Psychol.	10.1016/S0010-0285(03)00006-9	215
17	Binder J. R.	2000	Cereb. Cortex	10.1093/CERCOR/10.5.512	213
18	Hickok G	2004	Cognition	10.1016/J.COGNITION.2003.10.011	207
19	Lisker L	1964	Word	10.1080/00437956.1964.11659830	203
20	Scott S. K.	2000	Brain	10.1093/BRAIN/123.12.2400	203

* This analysis involved cited references and was not restricted to research articles.

On the other hand, Table 6 shows highly cited researchers as identified *via* citation analysis. For example, Naatanen R. (researcher 5) is well-recognised for his work on neurocognitive mechanisms underlying speech perception as revealed by the mismatch negativity (MMN), an auditory event-related brain potential that is elicited by discriminable changes in regular auditory input (Näätänen, 2001; Näätänen et al., 2005, 2007, 2014).

**Table 6:** Top 20 highly cited authors in speech perception studies.

IDs	Authors	Frequencies	IDs	Authors	Frequencies
1	Kuhl P. K.	1,994	11	Cutler A.	759
2	Werker J. F.	1,693	12	Zatorre R.J.	758
3	Hickok G.	1,433	13	Best C.T.	726
4	Liberman A. M.	1,347	14	Norris D.	713
5	Naatanen R.	1,268	15	Scott S.K.	664
6	Tallal P.	1,126	16	Bradlow A.R.	653
7	Boersma P.	872	17	Fowler C.A.	634
8	Flege J. E.	844	18	Pisoni D.B.	633
9	Nittrouer S.	790	19	Mcclelland J.L.	631
10	Jusczyk P. W.	784	20	Binder J.R.	604

A close look at the articles in Table 5 and some articles of researchers identified in Table 6, for example Werker J.F., Hickok G., Liberman A.M., reveals several important theoretical approaches to speech perception. Thus, we further synthesised the two tables to provide a theory-driven and coherent account of the results and, in doing so, highlighted some work or researchers that originally ranked low in each table.

First of all, the motor theory (Liberman et al., 1967), whose founder is identified as researcher 4 in Table 6, argues that the perceptual primitives in speech perception are not acoustic cues, but neural commands to articulators, or more recently, intended gestures in one’s mind (Liberman and Mattingly, 1985, research item 5 in Table 5). Listeners reconstruct talkers’ intended gestures, which are not susceptible to phonetic variations, from the speech signal. In this way, the motor theory naturally links speech perception and production and resolves the problem of lack of invariants between phonemes and acoustic signals.

An alternative theoretical approach to speech perception was proposed by Carol Fowler (see Table 6, researcher 17), that is the direct realist approach (Fowler, 1989; Best, 1995). The theory shares with the motor theory the view that perceptual targets are gestural in nature but argues that the actual gestures rather than the intended gestures are directly perceived. The gestural information correlates with acoustic patterns *via* the principles of acoustic physics. This implies that there is no need for mental representation and consequently no need for perceptual normalisation when dealing with variability in speech. In contrast to the motor theory which argues for a domain-specific mechanism, the direct realist approach contends that speech perception is domain-general, just like perception of other events in the world.

Apart from fundamental theoretical constructs *per se*, one essential theoretical issue from a developmental perspective is to account for how our efficiency in perceiving native language is obtained at the cost of losing sensitivities to speech sounds in other languages (Werker and Tees, 1984, research item 2 in Table 5 and researcher 2 in Table 6). One line of research that examines this issue aims at identifying the time point when babies are attuned by their ambient linguistic environments and lose sensitivity to non-native speech categories. For example, in one of the pioneering studies, 6-month-old infants in the United States and Sweden showed native language effects on speech perception (Kuhl et al., 1992).

Another line of research focuses on native language influences on perceiving non-native speech by adults. Several theoretical models (that account for such influences) and/or their founders were identified in Table 5 and/or Table 6. The Perceptual Assimilation Model (PAM: Best, 1995), as indicated in research item 13 in Table 5 and researcher 13 in Table 6, posits that adult listeners perceive non-native segments in an unfamiliar language according to their similarities to or discrepancies from the native gestural constellations that are closest to them in native phonological space. The model is built on the direct realist approach to speech perception and has been validated *via* a number of studies on non-native perception of consonants (Best et al., 2001), vowels (Tyler et al., 2014; Faris et al., 2018) and lexical tones (Chen et al., 2020).

The Speech Learning Model (SLM: Flege, 1995, research item 15 in Table 5 and researcher 8 in Table 6), accounts for native-language-induced perceptual difficulties *via* two mechanisms: (1) equivalence classification, in which distinct non-native phones are categorised to a single native category; (2) native language filtering, where features of non-native phones that are important phonetically but not phonologically, that is forming no meaningful contrasts, are filtered out.

A third model, the Native Language Magnet model (NLM: Kuhl, 1994; Kuhl and Iverson, 1995), whose founder ranked first in Table 6, posits that native language experience alters perceived distances in the acoustic space underlying phonetic categories and consequently influences non-native perception and production. The phonetic prototype, that is the ideal instance of a phonetic category, developed *via* native language experience, acts as a ‘perceptual magnet’ for other tokens in the category. It attracts those tokens towards itself by reducing the discrimination sensitivity as compared with non-prototypic tokens of the same category.

The most recent model is the Second Language Linguistic Perception model, or L2LP (Escudero, 2005), though relevant papers of which were not identified in Table 5 nor Table 6 because the two tables based on citations were biased towards earlier theories. Its founder, Paola Escudero, was among the top 20 most productive authors in Table 4. L2LP accounts for second language speech learning from the initial state to ultimate attainment. For the initial state, L2LP claims that non-native listeners rely on optimal perception, a perception grammar attuned by their native language. In other words, listeners initially perceive non-native phones in line with the acoustic features of their native language, called the Full Copying hypothesis. The model also contends that there is a direct link between perception and production and that perception of both native and non-native phones should match the acoustic properties of phones in participants’ native language/dialects (Escudero and Vasiliev, 2011).

These models all aim at explaining native language influences on non-native speech perception but with different theoretical foci. PAM, SLM and L2LP propose ways that non-native phones can be assimilated into native categories but only PAM and L2LP make explicit predictions concerning discrimination of contrasts based on assimilation patterns. SLM focuses more on perception and production of individual non-native phones, whereas NLM is concerned more about non-native phones that are assimilated as prototypical or non-prototypical tokens within a given native category and thus does not include predictions on contrasts of non-native phones that cross native phonological boundaries.

The evaluation of research impact based on total citations biases towards senior researchers and early publications. However, this fits our aim to find the theoretically foundational research and/or their founders. The identified theories are generally in line with findings of previous qualitative reviews (Diehl et al., 2004; Samuel, 2011; Antoniou, 2018), supporting the validity of our approach. Nonetheless, to compensate for the bias, we used citation network plots to visualise different citation relations, for example co-citation or bibliographic coupling, the latter of which reveals more about impactful emerging research.

**Figure 7:**
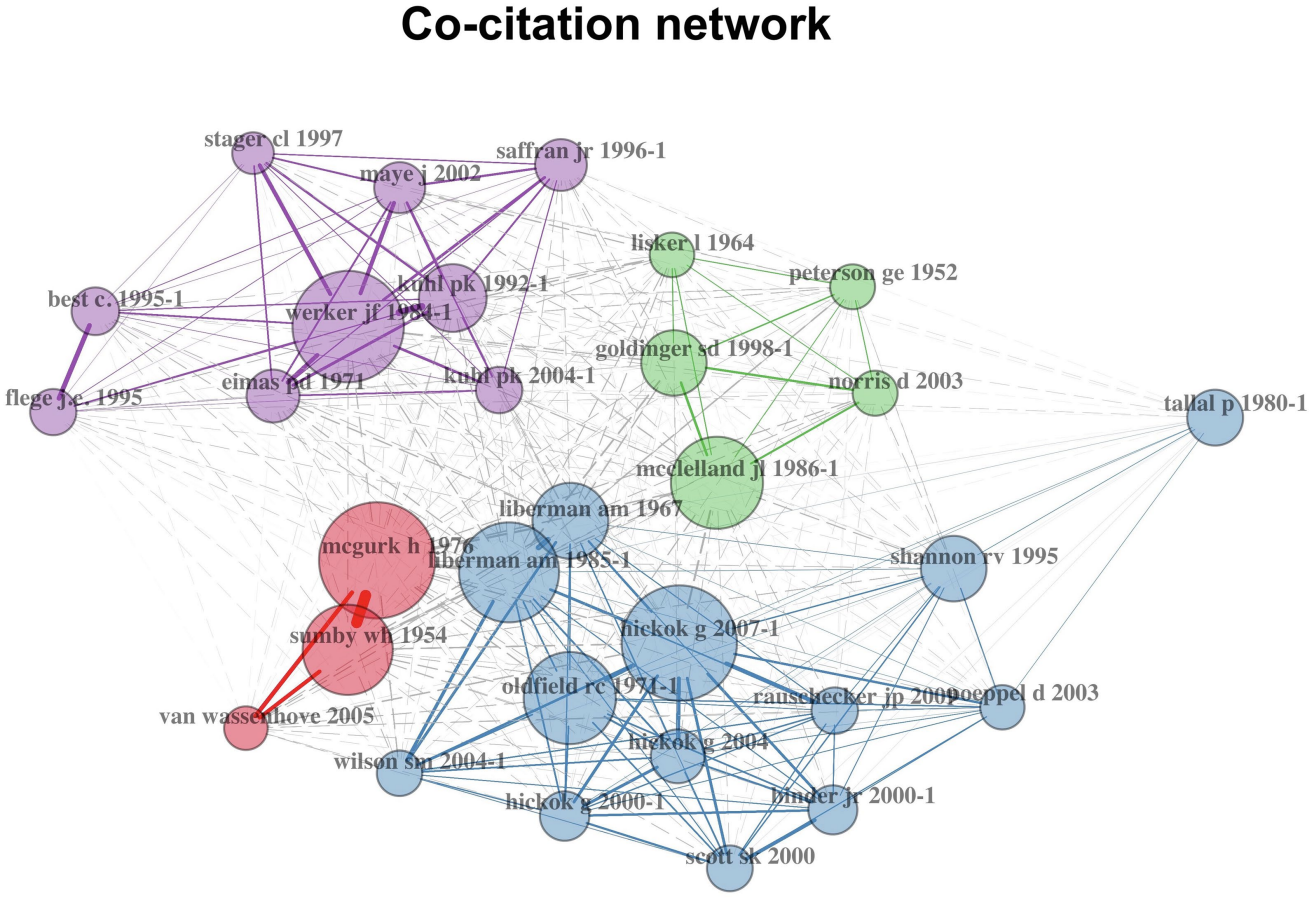
Co-citation network of speech perception research. Nodes stand for research articles. For each node, the label colour is the same as its cluster and its size is proportional to its co-citation degree.

When two articles are both cited in a third article, they are co-cited, forming a co-citation relation (Aria and Cuccurullo, 2017). Articles are co-cited when they contribute to similar or different aspects of a certain topic or paradigm. For example, in cross-language speech perception research, SLM, PAM, NLM and L2LP-related papers are often co-cited to lay out theoretical foundations and to form competing predictions. We constructed a co-citation network in which each node represented a research article and only the top 30 research articles were shown (Figure 7). The size of each node is proportional to the degree of co-citation. Clusters were found with the default algorithm in the *bibliometrix* package (Aria and Cuccurullo, 2017). Centrality statistics of each node can be found in the Supplementary Material  (Supplementary Table A).

Figure 7 illustrates co-citation relations in speech perception research. Four clusters were identified. First, research in the blue cluster was centred around the cortical organisation of speech processing. For example, the authors (Hickok and Poeppel, 2007, also identified as research item 4 in Table 5 and Hickok was identified as researcher 3 in Table 6), argued for a dual-stream model of speech processing, in which a ventral stream processes speech signals for comprehension and a dorsal stream maps acoustic speech signals to frontal lobe articulatory networks. The ventral stream is largely bilaterally organised whereas the dorsal stream is strongly left-hemisphere dominant.

Second, research in the green cluster was grouped around the TRACE model (McClelland and Elman, 1986), in which speech perception is simulated through excitatory and inhibitory interactions of processing units or ‘the Trace’ at feature, phoneme and word levels. In terms of perceptual targets, the model used acoustic dimensions of speech as its inputs, which is different from the motor theory and the direct realist approach. Unlike the two pure theoretical/psychological approaches, the TRACE model, representing a computation approach to the issue, considered both computational and psychological adequacy. That is the model can be programmed to recognise real speech and account for human performance in speech perception.

In addition, the red cluster revolved around audio-visual perception research (e.g., McGurk and Macdonald, 1976). The purple cluster of research focused on how native language development attunes speech perception (e.g., Werker and Tees, 1984).

**Figure 8:**
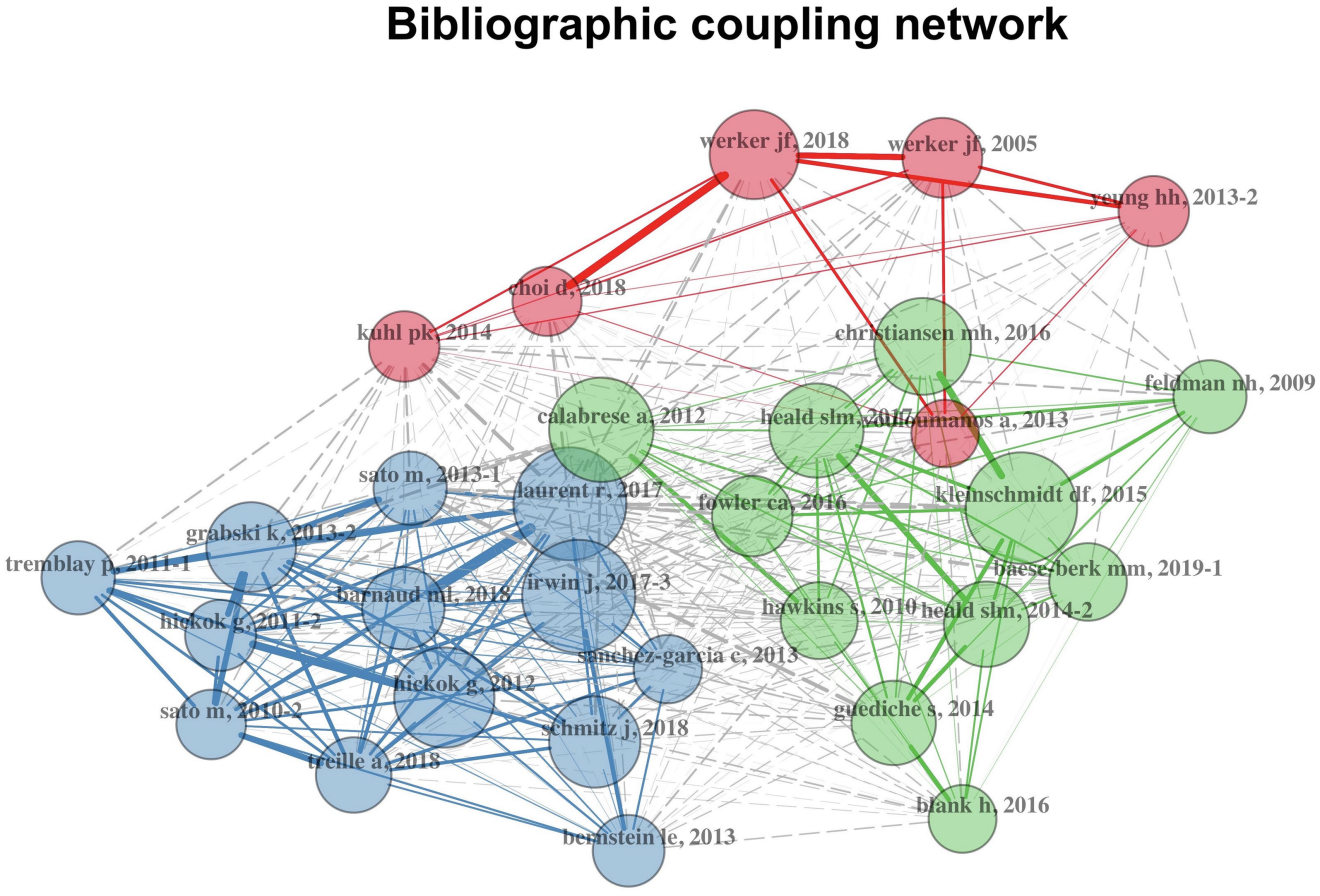
Bibliographic coupling network of speech perception research. Nodes stand for research articles. For each node, the label colour is the same as its cluster and its size is proportional to its bibliographic coupling degree.

Another citation relation is bibliographic coupling. Two articles are bibliographically coupled if at least one cited source appears in the references of both articles (Kessler, 1963). Some researchers argued that bibliographic coupling analysis better reflects unique information than co-citation when constructing citation networks (Kleminski et al., 2020). While co-citation analysis reveals more about relationships among older papers, bibliographic coupling analysis reflects more about the current research front. For this reason, the network (see Figure 8) based on bibliographic coupling relations was constructed here. Similar to the co-citation network, each node represented a research article and only the top 30 research articles were shown. The size of each node is proportional to the degree of bibliographic coupling (Aria and Cuccurullo, 2017). Clusters were found with the default algorithm in the *bibliometrix* package. Centrality statistics of each node can also be found in the Supplementary Material  (Supplementary Table B).

Three clusters of research were identified. First, research in the red cluster focused on developmental aspects of speech perception (Werker and Curtin, 2005; Choi et al., 2018; Werker, 2018). In the two review papers (Choi et al., 2018; Werker, 2018), the authors examined how infants’ universal sensitivities to human speech are attuned to their native language and specifically emphasised the multisensory nature of this process.

Second, research in the blue cohort concentrated on studies of cognitive processes in speech perception, such as audio-visual speech perception with application of the state-of-art brain science technologies (Sato et al., 2010; Irwin et al., 2017). For example, Irwin et al. (2017) tested typical developing children’s ability to detect the missing segment in spoken syllables with and without visual information and measured their behaviour and neural activities. They found that visual information attenuated the brain responses, suggesting that children were less sensitive to the missing segment when visual information of a face producing that segment was presented.

Third, research in the green cluster examined general speech perception issues, such as the lack of invariance problem (Kleinschmidt and Jaeger, 2015) or the perception–production link in speech learning (Baese-Berk, 2019).

Although co-citation and bibliographic coupling networks can reveal central research themes or topics, they require subjective interpretation of the clusters. To compensate for this disadvantage, term frequency analysis and co-word analysis were employed, the results of which can be directly interpreted according to the meanings of the words/phrases.

Assuming that terms with high frequency indicate important themes in the data set, term frequency can be used to identify important themes of a field (Khasseh et al., 2017; Zhao et al., 2018). We first analysed the frequency of author-defined keywords and Web of Science (WOS)-generated keywords.

In addition, bigram analysis, that is two-word combinations, was applied to the titles and abstracts of the articles. When extracting bigrams, we used the *unnest_tokens* function in the *tidytext* package (Silge and Robinson, 2016) in *R* and filtered package-defined stop words as they either occur in the first or second part of the bigram.

Table 7 shows the top 20 frequent terms. The most prominent research theme concerns speech perception in the clinical context with participants, such as children suffering from hearing loss or (developmental) dyslexia and the effects of cochlear implantation. Another line of research focuses on examining psycholinguistic issues, for example working memory, attention, spoken word recognition, as well as neurocognitive mechanisms underlying speech perception using brain science techniques, such as functional magnetic resonance imaging (fMRI) or electroencephalogram (EEG, including event-related brain potentials or ERPs).

**Table 7:** Top 20 terms from author-defined keywords, machine-generated keywords, title-based and abstract-based two-word phrases.

Author-defined		WOS-generated		Title		Abstract	
Terms	Freq	Terms	Freq	Terms	Freq	Terms	Freq
Cochlear Implant	243	Recognition	851	Cochlear implant	495	Hearing loss	950
fMRI	201	Discrimination	773	Audio-visual speech	175	Normal hearing	840
Dyslexia	162	Children	669	Hearing loss	155	Cochlear implant	587
Speech production	150	Information	539	Word recognition	132	Speech sounds	587
Children	146	Brain	429	Spoken word	97	Auditory cortex	541
Hearing loss	130	Noise	421	Auditory processing	93	Word recognition	535
Aging	117	Identification	408	Auditory cortex	91	Auditory processing	515
Development	106	Acquisition	387	Visual speech	91	Superior temporal	509
Phonology	104	English	376	Implant users	82	Speech production	494
Spoken word recognition	100	Integration	355	Event related	71	Visual speech	465
Auditory cortex	98	Intelligibility	340	Speech processing	70	Speech processing	460
Auditory perception	94	Infants	327	Developmental dyslexia	64	Speech recognition	448
Attention	93	Attention	321	Normal hearing	64	Native language	439
Auditory processing	92	Cortex	320	Word learning	64	Event related	427
Bilingualism	91	Comprehension	317	Language impairment	62	Cochlear implant	409
Audio-visual	89	Adults	313	Speech recognition	61	CI users	347
Language acquisition	87	Activation	312	Specific language	60	Phonological awareness	311
Multisensory	87	Hearing	311	Speech production	58	Audio-visual speech	305
Mismatch negativity	86	Working memory	304	fMRI study	53	Speech intelligibility	293
EEG	85	Age	296	Individual differences	53	Speech signal	289

Freq, Frequency.

**Figure 9:**
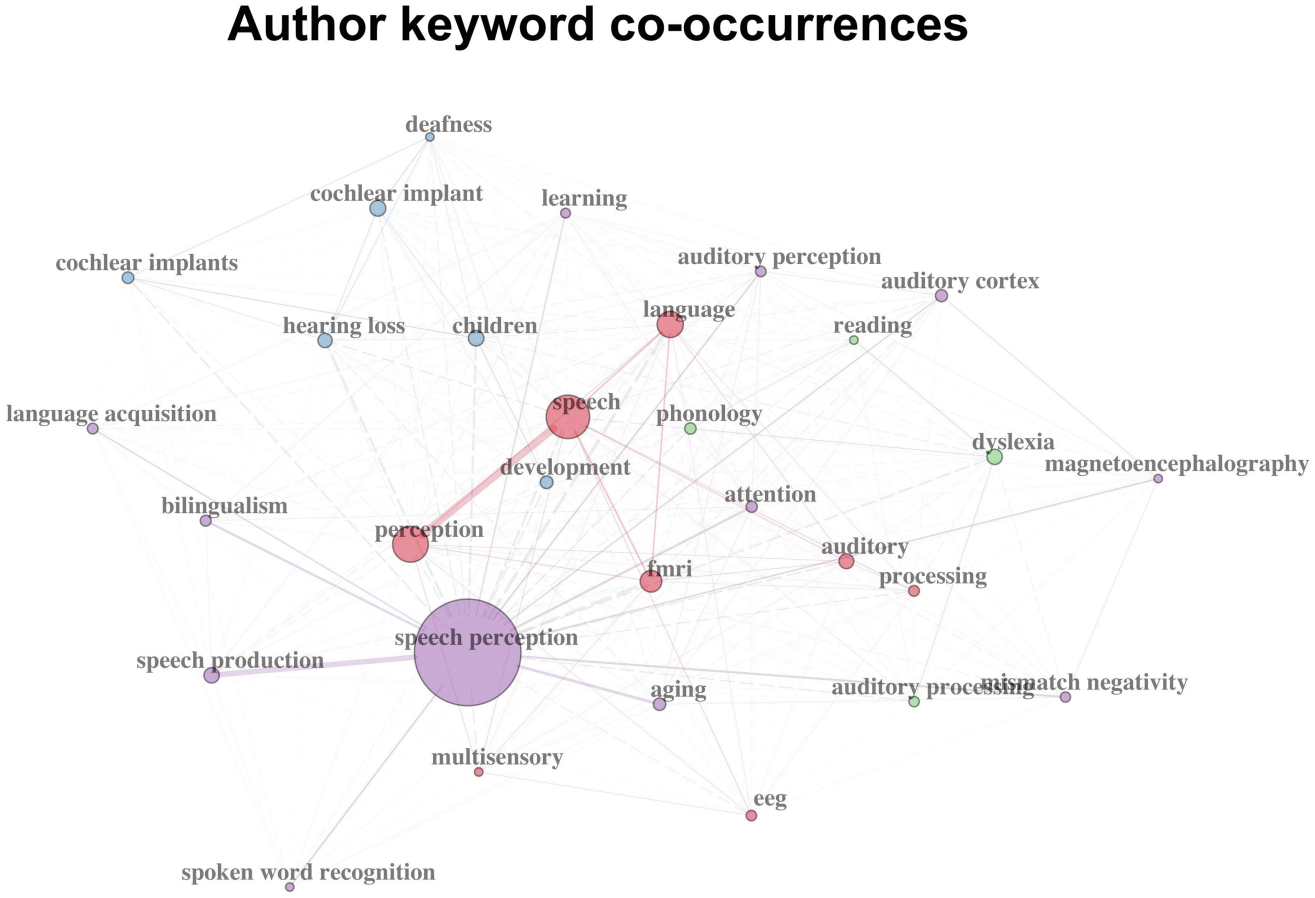
Author-defined keywords co-occurrence network.

In addition to term frequency, co-word networks were used to reveal both frequency and co-occurrence relationships among keywords. Co-word networks were constructed based on author-defined keywords (Figure 9). Keywords, such as cochlear implants or hearing loss, co-occur frequently with children, suggesting that this line of research focuses on hearing loss children in particular and examines the effect of cochlear implantation.

Another group of frequently co-occurring keywords again indicates the application of neuroscience technologies, such as EEG, fMRI or magnetoencephalography, in investigating brain activities during speech processing.

### General Discussion

In this section, we compare the findings of the present bibliometric review with previous qualitative reviews to provide a balanced discussion. First, our analysis revealed some important aspects of the field that were ignored by traditional qualitative reviews, such as major publication venues, research productivity and research collaboration.

Second, our analysis successfully detected major theoretical approaches in speech perception that were selected in previous qualitative reviews. These reviews featured in detailed descriptions of the theories with in-depth discussions of relevant empirical studies.

At the sublexical level, three theoretical approaches were introduced in two qualitative reviews (Diehl et al., 2004; Samuel, 2011), that is the motor theory, the direct realism and the general auditory approach, the former two of which were identified in our analysis. The third approach was not identified, presumably because it encompasses a number of theories in different publications (Diehl and Kluender, 1989; Ohala, 1996; Sussman et al., 1998). All of these theories shared that speech is perceived *via* the same auditory mechanisms involved in perceiving non-speech sounds and listeners recover messages from the acoustic signal without referencing it to articulatory gestures. We acknowledge that compared with experienced researchers in the field, the bibliometric analysis lacks the power of identifying certain research cohorts that used different terminologies for a similar theoretical approach.

In addition, Samuel (2011) discussed five phenomena/research paradigms relevant to foundational speech perception theories, that is categorical perception (Liberman et al., 1967), the right ear advantage (i.e., an advantage for perceiving speech played to the right ear, Shankweiler and Studdert-Kennedy, 1967), trading relations (i.e., multiple acoustic cues can trade off against each other in speech perception, Dorman et al., 1977), duplex perception (i.e., listeners perceive both non-speech and speech percept when acoustic cues of a speech sound are decomposed and presented separately to each ear, Rand, 1974) and compensation for coarticulation (i.e., perceptual boundaries are shifted to compensate for the coarticulation induced phonetic variations, Mann and Repp, 1981). These phenomena provided support or tests in the development of different theoretical approaches. Our analysis detected the categorical perception paradigm as it is closely related to the motor theory.

At the lexical and higher level, Samuel compared several models that account for how perceived phonetic features result in recognition of spoken words and/or utterances. All the models shared that speech perception involves activation of lexical representations *via* sublexical features, which was originally proposed by the Cohort model (Marslen-Wilson, 1975, 1987). However, they differed in whether the process is entirely bottom-up, as stated in the Merge model (Norris et al., 2000) and the fuzzy logical model (Massaro, 1989), or interactive, as maintained by the TRACE model (McClelland and Elman, 1986) and the Adaptive Resonance Theory (Grossberg, 1980). The TRACE model was identified in our co-citation analysis as an important research cohort and Norris D., who is the founder of a series of models including the Merge model, was identified in the citation analysis as an impactful researcher.

Three lexical-level phenomena were also discussed in Samuel’s review: the phonemic restoration effect (i.e., listeners’ ability to perceptually restore the missing phoneme, Warren, 1970), the McGurk effect (McGurk and Macdonald, 1976) and the Ganong effect (i.e., the bias of perceiving an ambiguous segment as a sound that forms a word, Ganong, 1980). In our analysis, we identified the original paper that reported the McGurk effect as an impactful research work and this topic as a research cohort.

Although Samuel’s review covered statistical learning and perceptual learning by infants and adults, the author did not discuss second language speech learning models. Another review with a focus on bilingual speech perception (Antoniou, 2018) discussed two speech learning models, that is SLM and PAM/PAM-L2. Both models were identified in our analysis, and we detected two additional models, NLM and L2LP.

It is important to reiterate here that our bibliometric review aims to complement, but not to replace, the existing qualitative reviews. Therefore, qualitative and bibliometric reviews should be combined to provide a comprehensive view of a field.

## Conclusion

In this study, a bibliometric analysis of journal articles published between 2000 and 2020 was conducted to map speech perception research. Major publication venues, general trends in research productivity, leading countries, research institutes and researchers were identified. Given that speech perception is an interdisciplinary field that requires research collaboration across traditional disciplinary boundaries, we did a research collaboration analysis, which indicated that researchers in the United States tended to collaborate more with researchers based in Canada, Spain, Japan and China, whereas geographically close European countries, such as the United Kingdom, France, Germany, Belgium, Switzerland, Northway and Italy, tended to collaborate with each other.

Analysis of research articles and researchers with high citations revealed three impactful theoretical approaches to speech perception, that is the motor theory, the direct realist approach and the computational approach, and four influential models that address key issues in non-native speech perception, that is SLM, PAM, NLM, and L2LP. Citation networks and term frequency analyses of keywords, titles and abstracts identified important research issues, such as cortical organisation of speech processing, audio-visual speech perception, spoken word recognition and infant/child speech learning.

Two directions for future research are (1) speech perception across the lifespan, for example infants and aging populations, or clinical populations, such as children with hearing loss or developmental dyslexia, and (2) application of neurocognitive techniques in understanding brain regions involved in speech perception and the time course of brain activities in speech processing.

## Data Availability Statement

The datasets presented in this study can be found in online repositories. The names of the repository/repositories and accession number(s) can be found at: https://osf.io/5hxg2/?view_only=42ccbe057ad84faca0e2560e4c0e36c3.

## Author Contributions

JC and HC: conceptualisation and writing—review and editing. JC: methodology, software, writing—original draft preparation, and visualisation. HC: supervision and funding acquisition. All authors contributed to the article and approved the submitted version.

## Conflict of Interest

The authors declare that the research was conducted in the absence of any commercial or financial relationships that could be construed as a potential conflict of interest.

## Publisher’s Note

All claims expressed in this article are solely those of the authors and do not necessarily represent those of their affiliated organizations, or those of the publisher, the editors and the reviewers. Any product that may be evaluated in this article, or claim that may be made by its manufacturer, is not guaranteed or endorsed by the publisher.

## Supplementary Material

The Supplementary Material for this article can be found online at: https://www.frontiersin.org/articles/10.3389/fpsyg. 2022.822241/full#supplementary-material

## Footnotes

^1^Some research that we analysed examined processing talker variability in speech to perceive linguistic categories but talker voice recognition, which is a related but different topic, is beyond the scope of this study.

^2^The Web of Science Core Collection provides full records, including cited references, which are essential for some bibliometric analysis, such as co-citation analysis. It has been widely used in conducting bibliometric analysis research. Other databases, which do not include full records or are not supported by the *bibliometrix* package, are not considered.
