# Supplementary material for: Sketching the Landscape of Speech Perception Research (2000–2020): A Bibliometric Study
Source: Front Psychol. 2022 Jun 2;13:822241. doi: 10.3389/fpsyg.2022.822241 (PMC9201966; doi:10.3389/fpsyg.2022.822241)
Supplement: Supplementary file 1 [file Data_Sheet_1.docx]

Supplementary Material

Table A. Centrality statistics of each node (vertex) in figure 6. Cluster column indicates the cluster each vertex belongs to. Cluster 1 = red; cluster 2 = blue; cluster 3 = green; cluster 4 = purple.

| Vertex | Cluster | Betweeness  centrality | Closeness  centrality | Pagerank  centrality |
| --- | --- | --- | --- | --- |
| mcgurk h 1976 | 1 | 0.216 | 0.034 | 0.052 |
| sumby wh 1954 | 1 | 0.195 | 0.034 | 0.044 |
| van wassenhove 2005 | 1 | 0.034 | 0.034 | 0.027 |
| liberman am 1967 | 2 | 0.418 | 0.034 | 0.048 |
| liberman am 1985-1 | 2 | 0.627 | 0.034 | 0.055 |
| oldfield rc 1971-1 | 2 | 0.302 | 0.034 | 0.035 |
| poeppel d 2003 | 2 | 0.120 | 0.033 | 0.023 |
| shannon rv 1995 | 2 | 0.227 | 0.034 | 0.023 |
| tallal p 1980-1 | 2 | 0.000 | 0.030 | 0.011 |
| hickok g 2004 | 2 | 0.141 | 0.034 | 0.036 |
| hickok g 2007-1 | 2 | 0.928 | 0.034 | 0.059 |
| rauschecker jp 2009 | 2 | 0.036 | 0.032 | 0.029 |
| wilson sm 2004-1 | 2 | 0.107 | 0.034 | 0.031 |
| scott sk 2000 | 2 | 0.066 | 0.033 | 0.032 |
| binder jr 2000-1 | 2 | 0.141 | 0.034 | 0.032 |
| hickok g 2000-1 | 2 | 0.139 | 0.034 | 0.033 |
| peterson ge 1952 | 3 | 0.090 | 0.034 | 0.022 |
| lisker l 1964 | 3 | 0.375 | 0.034 | 0.024 |
| mcclelland jl 1986-1 | 3 | 0.315 | 0.034 | 0.035 |
| goldinger sd 1998-1 | 3 | 0.061 | 0.033 | 0.029 |
| norris d 2003 | 3 | 0.116 | 0.034 | 0.024 |
| eimas pd 1971 | 4 | 0.107 | 0.034 | 0.033 |
| kuhl pk 1992-1 | 4 | 0.652 | 0.034 | 0.043 |
| werker jf 1984-1 | 4 | 0.729 | 0.034 | 0.057 |
| stager cl 1997 | 4 | 0.053 | 0.033 | 0.026 |
| maye j 2002 | 4 | 0.249 | 0.034 | 0.034 |
| best c. 1995-1 | 4 | 0.161 | 0.033 | 0.025 |
| kuhl pk 2004-1 | 4 | 0.275 | 0.034 | 0.026 |
| saffran jr 1996-1 | 4 | 0.113 | 0.034 | 0.026 |
| flege j.e. 1995 | 4 | 0.005 | 0.031 | 0.024 |

Table B. Centrality statistics of each node (vertex) in figure 7. Cluster column indicates the cluster each vertex belongs to. Cluster 1 = red; cluster 2 = blue; cluster 3 = green.

| Vertex | Cluster | Betweeness  centrality | Closeness  centrality | Pagerank  centrality |
| --- | --- | --- | --- | --- |
| werker jf, 2005 | 1 | 0.138 | 0.025 | 0.025 |
| choi d, 2018 | 1 | 3.223 | 0.034 | 0.028 |
| werker jf, 2018 | 1 | 0.141 | 0.026 | 0.030 |
| kuhl pk, 2014 | 1 | 2.898 | 0.033 | 0.030 |
| vouloumanos a, 2013 | 1 | 2.779 | 0.034 | 0.027 |
| yeung hh, 2013-2 | 1 | 0.199 | 0.024 | 0.020 |
| sato m, 2010-2 | 2 | 0.092 | 0.030 | 0.037 |
| barnaud ml, 2018 | 2 | 0.676 | 0.032 | 0.045 |
| treille a, 2018 | 2 | 0.030 | 0.029 | 0.037 |
| schmitz j, 2018 | 2 | 1.385 | 0.032 | 0.034 |
| laurent r, 2017 | 2 | 8.113 | 0.034 | 0.058 |
| irwin j, 2017-3 | 2 | 2.699 | 0.034 | 0.038 |
| sato m, 2013-1 | 2 | 0.720 | 0.033 | 0.036 |
| grabski k, 2013-2 | 2 | 0.121 | 0.030 | 0.046 |
| sanchez-garcia c, 2013 | 2 | 2.228 | 0.034 | 0.032 |
| bernstein le, 2013 | 2 | 0.178 | 0.031 | 0.027 |
| hickok g, 2012 | 2 | 0.235 | 0.031 | 0.039 |
| tremblay p, 2011-1 | 2 | 0.058 | 0.030 | 0.032 |
| hickok g, 2011-2 | 2 | 0.064 | 0.030 | 0.039 |
| hawkins s, 2010 | 3 | 0.709 | 0.034 | 0.030 |
| feldman nh, 2009 | 3 | 0.843 | 0.029 | 0.025 |
| baese-berk mm, 2019-1 | 3 | 1.091 | 0.034 | 0.026 |
| heald slm, 2017 | 3 | 2.008 | 0.034 | 0.036 |
| blank h, 2016 | 3 | 0.080 | 0.030 | 0.024 |
| fowler ca, 2016 | 3 | 0.705 | 0.033 | 0.030 |
| christiansen mh, 2016 | 3 | 1.040 | 0.033 | 0.028 |
| kleinschmidt df, 2015 | 3 | 1.538 | 0.034 | 0.038 |
| heald slm, 2014-2 | 3 | 1.494 | 0.034 | 0.033 |
| guediche s, 2014 | 3 | 0.218 | 0.031 | 0.033 |
| calabrese a, 2012 | 3 | 4.296 | 0.034 | 0.040 |
